# Plasmacytoid dendritic cell biology and its role in immune‐mediated diseases

**DOI:** 10.1002/cti2.1139

**Published:** 2020-05-26

**Authors:** Yishan Ye, Béatrice Gaugler, Mohamad Mohty, Florent Malard

**Affiliations:** ^1^ INSERM, Centre de Recherche Saint‐Antoine (CRSA) Sorbonne Université Paris France; ^2^ Bone Marrow Transplantation Center The First Affiliated Hospital School of Medicine Zhejiang University Hangzhou China; ^3^ Service d’Hématologie Clinique et Thérapie Cellulaire AP‐HP, Hôpital Saint‐Antoine Sorbonne Université Paris France

**Keywords:** alloreactivity, autoimmunity, cell development, immunotherapy, plasmacytoid dendritic cells

## Abstract

Plasmacytoid dendritic cells (pDCs) are a unique subset of dendritic cells specialised in secreting high levels of type I interferons. pDCs play a crucial role in antiviral immunity and have been implicated in the initiation and development of many autoimmune and inflammatory diseases. This review summarises the latest advances in recent years in several aspects of pDC biology, with special focus on pDC heterogeneity, pDC development via the lymphoid pathway, and newly identified proteins/pathways involved in pDC trafficking, nucleic acid sensing and interferon production. Finally, we also highlight the current understanding of pDC involvement in autoimmunity and alloreactivity, and opportunities for pDC‐targeting therapies in these diseases. These new insights have contributed to answers to several fundamental questions remaining in pDC biology and may pave the way to successful pDC‐targeting therapy in the future.

## Graphical abstract

This review summarises the latest advances in several aspects of plasmacytoid dendritic cell (pDC) biology, with a special focus on pDC heterogeneity, pDC development via the lymphoid pathway, and newly identified proteins/pathways involved in pDC trafficking, nucleic acid sensing and interferon production. We also highlight the current understanding of pDC involvement in autoimmunity and alloreactivity, and opportunities for pDC‐targeting therapies in these diseases.

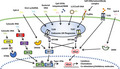

## Introduction

Human plasmacytoid dendritic cells (pDCs) were initially described 20 years ago by the Liu and Colonna groups.^1^, ^2^ pDCs are continuously generated from haematopoietic stem cells in the bone marrow (BM) via both myeloid and lymphoid precursors. Afterwards, proteins such as CXCR4 context‐dependently mediate the trafficking of pDCs from the BM to peripheral blood and subsequent migration to specific target tissues.

Plasmacytoid dendritic cells constitute 0.1–0.5% of human peripheral blood mononuclear cells (PBMCs).^3^, ^4^ Freshly purified pDCs manifest a plasmacytoid morphology, with rough endoplasmic reticulum and Golgi apparatus. Upon activation, pDCs gain dendritic cell‐like morphology and produce massive amounts of type I interferons (IFN‐I), for example most of the IFN‐I detectable in the blood following viral infection in mice and humans.^1^, ^2^ The IFN‐I secretion by pDCs is mainly mediated through the activation of the endosomal Toll‐like receptors (TLRs) TLR7 and TLR9, with cytosolic receptor initiating pathways playing an important supplementary role.^5^ Apart from IFN‐I, pDCs could also secrete pro‐inflammatory cytokines and chemokines and express co‐stimulatory or co‐inhibitory molecules which facilitate pDCs to cross‐prime CD8^+^ T cells and present antigens to CD4^+^ T cells.^2^, ^6^

Plasmacytoid dendritic cells have been shown to be implicated in many autoimmune diseases such as systemic lupus erythematosus (SLE) and systemic sclerosis (SSc).^7^, ^8^ Furthermore, in graft‐versus‐host disease (GVHD), a major immunologic complication after allogeneic haematopoietic cell transplantation (allo‐HCT), our group and others have identified an important role of pDCs during disease occurrence and development.^9^, ^10^ Based on these observations, several pDC‐targeting drugs such as anti‐interferon‐α (anti‐IFN‐α) monoclonal antibody (mAb) and anti‐type I IFN receptor subunit‐1 (anti‐IFNAR1) mAb are being assessed in SLE in clinical trials and have shown promising outcomes.^11^, ^12^, ^13^

This review summarises the recent advances in pDC biology, including pDC heterogeneity, lymphoid pathway of pDC development and novel nucleic acid sensing patterns during IFN‐I production. Furthermore, the newly identified roles of pDC in immune‐mediated diseases and novel pDC‐targeting drugs assessed in this setting are also described.

## Definition of pDCs

Human pDCs were traditionally defined as not expressing the lineage‐associated markers (Lin) CD3, CD19, CD14, CD16 and CD11c, but selectively expressing CD303 (BDCA2), CD304 (BDCA4) and immunoglobulin‐like transcript 7 (ILT7).^14^ They also express CD4, CD45RA, CD68, ILT3 and CD123 (IL‐3 receptor α‐subunit). Mouse pDCs were identified with CD11c, CD45RA, B220, Ly6C, bone marrow stromal antigen 2 (BST2; also known as tetherin) and sialic acid‐binding immunoglobulin‐like lectin H (Siglec‐H).^15^ However, a specific subset of CD2^hi^CD5^+^CD81^+^ human pDCs was later identified, which express the pDC markers CD123, CD303 and CD304, but do not secrete IFN‐I. Meanwhile, upon activation, they secrete IL‐12 and potently prime T‐ and B‐cell responses.^16^, ^17^, ^18^ Recently, this non‐canonical ‘pDC subset’ has been redefined as Axl^+^ DCs with the development of single‐cell analysis, which has divided human DCs into 6 putative subsets, namely cDC1, cDC2‐A, cDC2‐B, CD16^+^ DC, pDC and Axl^+^ DC (reviewed by Rhodes *et al*. 2019).^19^, ^20^, ^21^, ^22^ Accordingly, the traditionally defined pDCs include two distinct subsets, the canonical IFN‐I‐producing pDCs (referred to as ‘canonical pDC’ hereafter) and the Axl^+^ DCs, which are inefficient at IFN‐I production but can efficiently activate T/B cells.^22^ The Axl^+^ DCs are a distinct myeloid DC population expressing typical markers Axl and Siglec6 and are a continuum of pDC and cDC2 characteristics. There is considerable diversity within Axl^+^ DCs, ranging from the pDC‐like state expressing typical pDC markers (CD123, BDCA2, BDCA4, CD45RA) to the cDC2‐like state expressing typical cDC2 markers (CD11c, CD33, CX3CR1, CD1c, CD2).^21^, ^22^ Meanwhile, from the pDC‐like state to the cDC2‐like state, there is a decreased expression of TCF4 and an increased expression of ID2, the signature transcription factors for pDC and cDC2, respectively.^21^, ^22^ Moreover, the murine counterpart of Axl^+^ DCs with identical genetic and functional characteristics is also identified.^23^, ^24^

The canonical pDC population was initially regarded as ‘bona fide’ IFN‐I‐producing cells without antigen‐presenting capacity.^20^ Later, studies revealed that upon activation with IL‐3 and CD40L, influenza virus or oligodeoxyribonucleotides with CpG motifs (CpG ODNs), canonical pDCs show enforced T‐cell activation capacity by expressing higher levels of co‐stimulatory molecules/chemokine receptors and lower levels of co‐inhibitory molecules.^22^, ^25^ Furthermore, heterogeneity of canonical pDC has started to be investigated. Alculumbre *et al.* ^25^ observed that canonical pDCs could be divided into three relatively stable subsets depending on their CD80 and PD‐L1 expression. The P1‐pDCs (PD‐L1^+^CD80^–^) have a plasmacytoid morphology and are specialised in IFN‐I production. The P3‐pDCs (PD‐L1^−^CD80^+^) have a dendritic morphology and are more potent in T‐cell activation. Finally, the P2‐pDCs (PD‐L1^+^CD80^+^) display a phenotype and morphology between the P1‐ and P3‐pDCs.^25^

Given their recent discovery, the ontogeny and immune functions of Axl^+^ DCs remain to be elucidated. Therefore, in the following sections of this review, ‘pDC’ means traditionally defined pDCs unless otherwise indicated. Collectively, the identification of Axl^+^ DCs and the heterogeneity of canonical pDCs have prompted us to re‐evaluate pDC development and several important aspects of pDC biology.

## Development of pDCs

**Figure 1:**
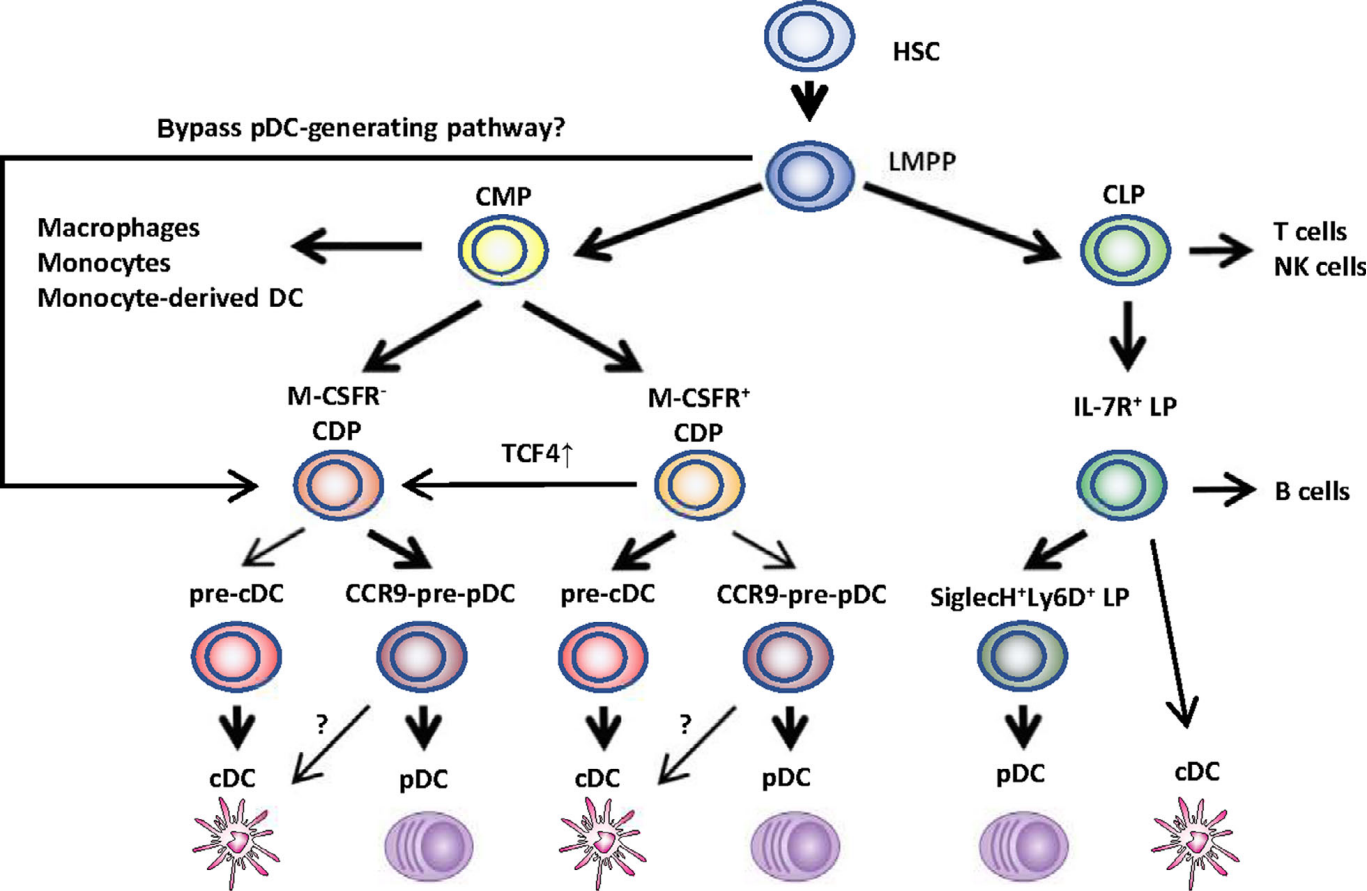
Developmental pathways of pDCs. Major (heavy arrows) and minor (light arrows) haematopoietic pathways found to have the potential to produce plasmacytoid dendritic cells (pDCs) or conventional dendritic cells (cDC) are outlined. The progenitors include the following: HSS, haematopoietic stem cells; LMPP, lymphocyte primed multipotent precursors; CMP, common myeloid precursors; CLP, common lymphoid precursors; CDP, common dendritic precursors; LP, lymphoid precursors; pre‐cDC, precursors of cDC; and pre‐pDC, precursors of pDC. It is not yet clear whether a proportion of M‐CSFR^‐^ CDP could be derived from LMPP via a more direct ‘bypass pathway’. CCR9^−^ pre‐pDC could differentiate into ‘cDC‐like’ cells context‐dependently, while these cells are not yet identified as real cDCs.

The development of pDCs is schematically shown in Figure 1. pDCs are continuously generated from haematopoietic stem cells in the BM *via* both myeloid and lymphoid pathways. Flt3 and its ligand Flt3L are crucial for pDC development in the mouse and human.^26^, ^27^ The other important cytokine promoting pDC development is M‐CSF (encoded by *csf‐1*), which is able to drive pDCs and cDCs from BM precursor cells *in vitro* and *in vivo*.^28^ The pDC transcription programme seems to initiate from progenitors expressing IRF8.^29^ The specific development of pDCs requires the transcription factor TCF4, as shown in murine pDCs.^30^ As the master regulator, TCF4 acts with its transcription co‐factors including SPIB, IRF8 and RUNX2, among others, which are involved in the development, homoeostasis and function of pDCs.^30^, ^31^, ^32^

Within the myeloid pathway, the common myeloid progenitors develop firstly into earlier precursors named myeloid precursors, which subsequently differentiate into macrophages and DC precursors (MDPs). Murine MDPs are Lin^−^CX3CR1^+^CD11b^−^c‐Kit^hi^Flt3^+^ and macrophage colony‐stimulating factor receptor (M‐CSFR or CD115) positive. Finally, MDPs give rise to monocytes and common DC progenitors.^33^ CDPs express Lin^−^c‐Kit^int/lo^Flt3^+^IL‐7R^−^ and comprise M‐CSFR‐positive (M‐CSFR^+^ CDPs) and M‐CSFR‐negative (M‐CSFR^−^ CDPs) subsets, which preferentially give rise to cDCs and pDCs, respectively.^34^ M‐CSFR^−^ CDPs express a high level of TCF4 (E2‐2), while TCF4 expression on M‐CSFR^+^ CDPs is low.

A pDC progenitor close to terminal differentiation was identified in mice, which shares most properties with mature pDCs, but does not express CCR9, and expresses low class II major histocompatibility complex (MHC II).^35^ CCR9^−^ pDC progenitors account for about 20% of murine BM pDCs. They could migrate into peripheral organs and undergo tissue‐specific differentiation into either terminal CCR9^+^ pDCs or cDC‐like cells.^36^ The plasticity of this CCR9^−^ pDC progenitor indicates that the conversion of pDC to cDC could happen close to terminal differentiation.

The lymphoid origin of pDCs was proposed soon after their identification, with the observation that both common myeloid progenitors and common lymphoid progenitors had the potential to produce pDCs after transfer into irradiated mice.^37^ Moreover, murine pDCs of lymphoid origin showed evidence of past recombination activating gene 1 (RAG1) expression and had D‐J rearrangements in IgH genes, which are gene arrangement processes normally restricted to lymphoid lineage cells.^26^ Recently, a pDC progenitor within the IL‐7R^+^ lymphoid precursors was identified in mice. IL‐7R^+^ lymphoid precursors could differentiate into both pDCs and B cells, with the specific subset of SiglecH^+^Ly6D^+^‐double‐positive subset giving rise exclusively to pDCs when cultured in the presence of Flt3L.^38^ Similarly, a common IL‐7R^+^ progenitor of both pDCs and B cells has also been identified in humans.^39^

It was initially considered that the majority of pDCs derive from myeloid progenitors, with the evidence that the majority (~80%) of pDCs became labelled with *in vivo* lineage tracing using the common DC progenitor (myeloid origin) marker Csf1r.^40^ Moreover, progenitors with transcriptomic features of pDCs emerge before lymphoid progenitors^29^ and pDCs develop from stem cells *in vivo* with the same kinetics as myeloid cells including cDCs.^41^ This theory is, however, challenged with new findings. Rodrigues *et al.* observed that murine mature BM and splenic pDCs differentiate *in vitro* and *in vivo* predominantly from IL‐7R^+^ lymphoid progenitors. Further single‐cell analysis revealed that mature pDC subsets derived from both myeloid and lymphoid origins are able to secrete IFN‐I, but only myeloid‐derived pDCs share with cDCs the ability to process and present antigen.^38^ Given that Axl^+^ DCs were not excluded in this study, these ‘myeloid‐derived pDCs’ may represent the Axl^+^ DCs and/or the P3‐pDCs (PD‐L1^−^CD80^+^).

Importantly, a series of studies have warranted ‘revisiting’ the DC progenitors previously defined solely by phenotype. Sathe *et al.* ^26^ observed that murine pDCs derived from the Lin^−^c‐Kit^−^sca‐1^−^ MDPs showed ‘lymphoid’ characteristics of past RAG1 expression and had D‐J IgH gene rearrangements, indicating a possible pDC lineage imprinting in earlier progenitors. In addition, murine MDPs were found to contain predominantly precursors of macrophages/monocytes but few precursors of resident pDCs, thus challenging MDPs as the major source of myeloid pDCs.^42^ Indeed, recent studies have observed that several progenitors, such as the lymphocyte primed multipotent progenitor, are heterogeneous at the clonal level and include progenitors of many different functional potentials.^43^ In summary, pDCs derive from both myeloid and lymphoid pathways, and the exact programme for pDC lineage imprinting remains to be elucidated, which may occur in earlier haematopoietic progenitors.^44^ Further comprehensive studies on the transcriptomic programme are crucial to better trace the fate of pDCs.

## Trafficking of pDCs

Plasmacytoid dendritic cells are constantly produced in the BM and migrate to the primary and secondary lymphoid organs via peripheral blood during homoeostasis. Human and murine pDCs constitutively express CXCR4, and the CXCR4–CXCL12 signalling is crucial for the early development of pDCs within the BM stromal cell niches, and their migration towards splenic white pulp.^3^, ^4^ Circulating pDCs migrate from the blood compartment into lymph nodes mainly through high endothelial venules in both humans and mice.^2^, ^15^ pDCs constitutively express high levels of L‐selectin,^15^ CXCR4^4^ and ChemR23,^45^ whose ligands are expressed by high endothelial venules. Therefore, these proteins are responsible for pDC trafficking to lymph nodes during homoeostasis. In addition, chemokine receptors including CCR2, CCR5, CCR6, CCR7, CCR9 and CCR10 are expressed on pDCs and facilitate the homing to peripheral blood during homoeostasis or inflammation (reviewed by Swiecki and Colonna 2015).^46^ Moreover, during inflammation, additional molecules are involved in pDCs homing to lymph nodes, such as PSGL‐1, the ligand for E‐selectin, β1 and β2 integrins and CXCR3.^46^ Other proteins involved in pDC migration and organ localisation include MAdCAM‐1^47^ and IFN‐β.^48^

In addition to receptors expressed on the surface, several intracellular signalling molecules have been identified as playing a decisive role in pDC migration. CD2‐associated protein, which is specifically expressed in human and murine pDCs, is correlated with pDCs’ lymph node migration under conditions of inflammation in mice.^49^ Moreover, dedicator of cytokinesis protein 2 (DOCK2) is found to be indispensable for migration of murine pDCs.^50^

## Nucleic acid sensing and IFN secretion by pDCs

Plasmacytoid dendritic cells were initially identified as a unique cell subset that respond to viruses with rapid and massive production of IFN‐I, and play a central role in the antiviral immune response.^2^ Moreover, pDCs could also respond to certain non‐viral pathogens such as bacteria (e.g. Chlamydia pneumoniae)^51^ and apicomplexan parasites (e.g. Plasmodium).^52^, ^53^ The recent advances in pDCs in anti‐infectious immunity are not within the scope of this review, but have been very well summarised by Reizis.^54^ Recognition of either pathogen‐derived nucleic acids or synthetic TLR ligands such as CpG ODNs initiates IFN‐I secretion by pDCs, which is mainly (albeit not exclusively) mediated through the activation of the endosomal TLR7 and TLR9, and the subsequent myeloid differentiation primary response protein 88 (MYD88)–interferon regulatory factor 7 (IRF7) pathway.^55^ In addition, the MYD88–NF‐κB pathway is also activated, leading to the secretion of pro‐inflammatory cytokines and chemokines, and the expression of co‐stimulatory molecules. TLR7 senses RNA viruses and endogenous RNA, whereas TLR9 detects prokaryotes containing unmethylated CpG‐rich DNA sequences and endogenous DNA. Both TLR7 and TLR9 sense synthetic CpG ODNs, and different classes of CpG ODNs have been developed to perform different immune functions. CpG‐A is a strong inducer of type I IFNs, whereas CpG‐B is a potent stimulator of maturation and the production of cytokines and chemokines. CpG‐C exhibits properties of both CpG‐A and CpG‐B.^56^

Most cell types other than pDCs constitutively express IRF3, but not IRF7 or only at a very low level. Upon viral infection, IFN‐β can be directly induced by IRF3 and promotes both their own secretion and that of IFN‐α in an autocrine manner mediated by type I IFN receptor (IFNAR).^57^ This IFNAR‐based feedback signalling is crucial for the massive production of IFN‐I during viral infection. Notably, pDCs constitutively express higher levels of IRF7 than do other cell types,^58^ and are able to secrete IFN‐I rapidly and independently of the IFN‐I receptor IFNAR‐based feedback signalling.^59^ Consistently, studies have shown that IFNAR is dispensable for pDCs during certain virus infections *in vivo* including vesicular stomatitis virus (VSV)^59^ and mouse cytomegalovirus (MCMV).^60^ However, the ultimate IFN‐I responses by pDCs to TLR ligands *in vivo* ^61^ or to certain viruses *in vitro* ^62^ require IFNAR signalling, iing the necessity for intact IFNAR‐based feedback for optimal pDC function.

**Figure 2:**
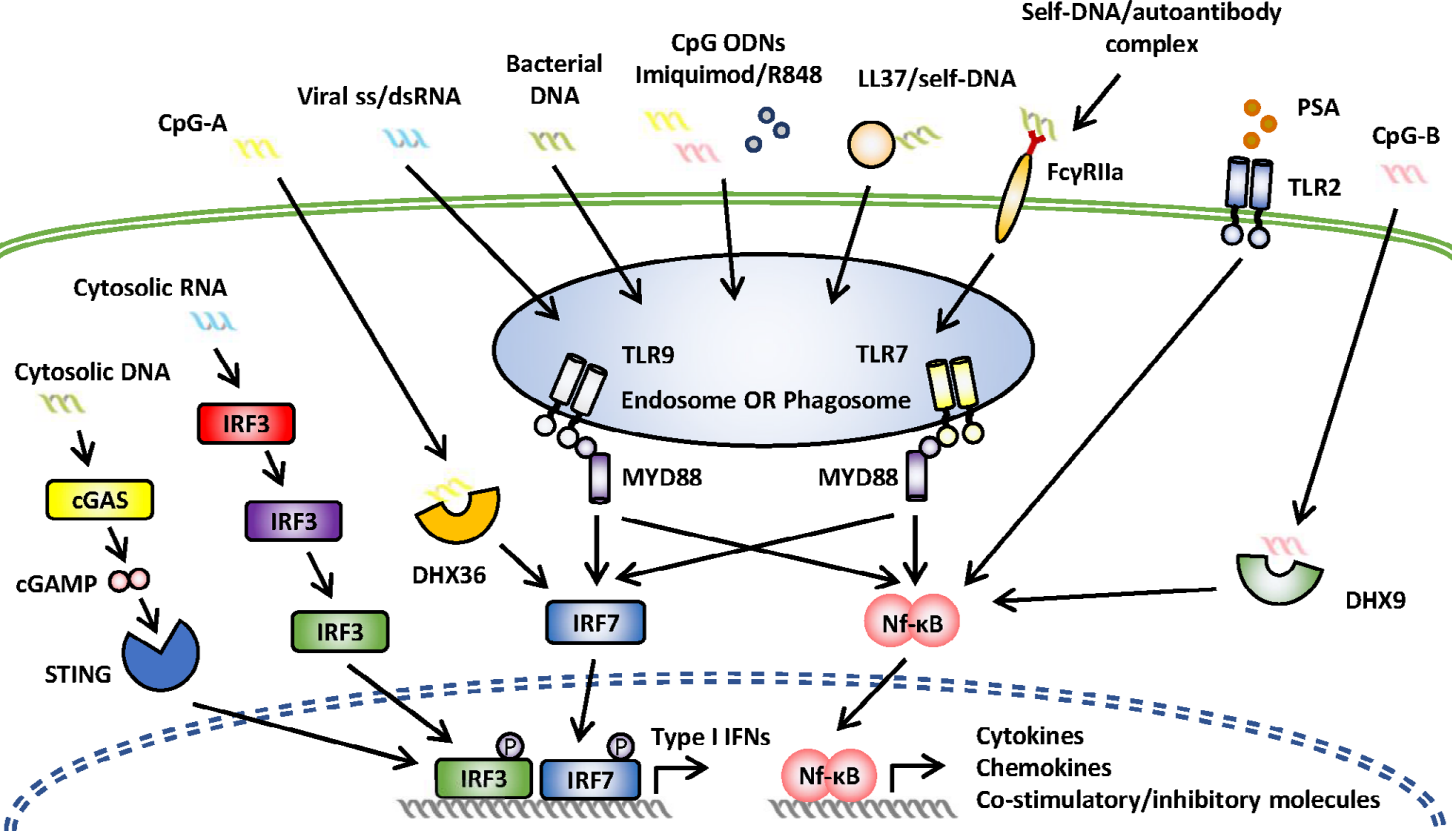
Routes of pDC sensing. *Endosomal pathways*: TLR7 senses RNA viruses and endogenous RNA, whereas TLR9 detects prokaryotes containing unmethylated CpG‐rich DNA sequences and endogenous DNA. Both TLR7 and TLR9 sense synthetic TLR ligands (CpG ODNs/imiquimod/R848) and immune complexes (self‐DNA/autoantibody and LL37/self‐DNA complexes mediated by FcγIIa). *Non‐endosomal pathways*: The cGAS (cyclic GMP‐AMP (cGAMP) synthase)–STING (stimulator of interferon genes) pathway senses cytosolic DNA and triggers an IRF3‐mediated IFN‐I production. Retinoic acid‐inducible gene I (RIG‐I) senses replicate viral RNA, recruits the mitochondrial antiviral signalling protein adaptor protein and leads to IFN‐I production. (DExD/H)‐box helicases DHX36 and DHX9 sense CpG ODNs, with the former selectively binding to CpG‐A and activating the IRF7 pathway and the latter selectively binding CpG‐B and activating the Nf‐ĸB pathway. pDCs sense polysaccharide A (PSA) via cytosolic TLR2 and activate the Nf‐ĸB pathway.

Not long after its identification, the TLR‐mediated sensing of pDCs was found to be not exclusive with the observation that pDCs could generate IFN‐α in response to the DNA virus herpes simplex virus type 1 (HSV‐1) independent of TLR9 signalling.^63^ Gradually, alternative sensing systems initiated by cytosolic receptors were revealed. Human pDCs could sense cytosolic DNA via the cGAS (cyclic GMP‐AMP (cGAMP) synthase)–STING (stimulator of interferon genes) pathway, which thereby triggers an IRF3‐mediated IFN‐I production independent of TLR9.^5^ Moreover, both human and murine pDCs express the cytosolic RNA sensor retinoic acid‐inducible gene I (RIG‐I), which senses replicate viral RNA, recruits the mitochondrial antiviral signalling protein adaptor protein and finally leads to IFN‐I production.^62^ Other cytosolic sensors include (DExD/H)‐box helicases DHX36 and DHX9 expressed on human pDCs, with the former selectively binding to CpG‐A and activating the IRF7 pathway and the latter selectively binding to CpG‐B, leading to subsequent activation of the Nf‐ĸB pathway.^64^ Collectively, these cytosolic receptor initiating pathways may play an important supplementary role in pDC immunity. Routes of pDC sensing are summarised in Figure 2.

Despite their low frequency, pDCs produce most of the IFN‐I detectable in the blood following viral infection. Meanwhile, upon *in vivo* CpG ODN activation in mice, the IFN‐I response is mediated exclusively by pDCs.^65^ Given that TLR7 and TLR9 are also expressed on B cells and several myeloid cell types, an important question is raised: Why and how pDCs, but not other cell types, activate this signalling pathway for IFN‐I induction? So far, it seems that a combination of cellular processes contributes to the answer to this question. Firstly, CpG‐A is retained for long periods in the early endosome of pDCs, together with the MYD88–IRF7 complex, whereas in cDCs, CpG‐A is quickly transferred to lysosomal vesicles.^66^, ^67^ Moreover, protein kinase C and casein kinase substrate in neurons 1 (PACSIN1) is specifically expressed on human and mouse pDCs and is involved in the type I IFN, but not the pro‐inflammatory cytokine secretion in response to the TLR9 ligand.^68^

Given that both the IRF7 and NF‐κB pathways depend on MYD88 and UNC93B, why and how pDCs ‘select’ the IRF7 pathway to secrete IFN‐I has been intensively investigated. The compartment in which TLRs encounter their ligands seems to be the decisive factor.^67^ Another important factor mediating the preferential secretion of IFN‐I is the adapter protein‐3 (AP3).^69^ The AP3 adaptor complex and the AP‐3‐interacting cation transporter Slc15a4 are responsible for the trafficking of TLR9 from the early endosome to a specialised lysosome‐related organelle (IRF7 endosome), where TLR9 activates the MYD88 signalling this IFN‐I secretion.^70^ In addition, a non‐canonical recognition process called microtubule‐associated protein 1A/1B‐light chain 3 (LC3)‐associated phagocytosis (LAP) was identified when pDCs were exposed to large DNA containing immune complexes.^71^ It was recently found that LAP is also involved in CpG ODN‐induced TLR9 sensing.^72^

Plasmacytoid dendritic cells produce high levels of IFN‐I during MCMV infection *in vivo* through the TLR9–MYD88–IRF7 signalling pathway. Surprisingly, this process is dependent on neither AP3‐driven endosomal routing nor the autophagy‐related 5 (Atg5)‐dependent LAP, indicating a potentially unknown mechanism involved in TLR sensing.^60^

Apart from the cell‐intrinsic mechanism for type I interferon production, recent studies have indicated the involvement of a cooperative mechanism. It was previously observed that *in vivo* pDC activation by TLR ligands induced their tight clustering.^61^ *In vitro*, CpG ODN‐activated human pDCs produce higher IFN‐I when cultured with high cell density, which was proved in a single‐cell activation assay.^73^ This phenomenon is partly explained by an autocrine/paracrine mechanism. Moreover, recent studies reveal that cell–cell contacts may also contribute to the enhanced IFN‐I secretion within clustered pDCs.^60^, ^74^, ^75^ Lymphocyte function‐associated antigen 1 (LFA‐1) is found to be responsible for cell–cell contact of pDCs in humans^74^ and mice^75^ *in vitro*. Saitoh *et al.* ^75^ observed that murine pDCs lacking LFA‐1 have decreased IFN‐I production in response to TLR ligands and to the influenza virus, which is due to impaired intracellular TLR7 trafficking to the cell–cell contacts and subsequent IFN‐I secretion in the vicinity. Moreover, the optimal *in vivo* activation of IFN‐I production by murine pDCs during MCMV infection or TLR9 ligand activation also requires LFA‐1 expression.^60^

The cooperative mechanism also plays a crucial role in virus sensing. pDCs could respond efficiently to viruses (e.g. influenza virus) without being infected, through internalised virions which initiate the IFN‐I response.^2^ However, some viruses (e.g. VSV) only drive the IFN‐I response when replicate‐active. In these cases, cooperative virus sensing would happen between uninfected pDCs and infected pDCs, or between uninfected pDCs and infected cells other than pDCs. The homotypic interaction was supported by studies showing that during certain virus infections (including VSV), the viruses replicated in a certain subset of pDCs while substantial IFN‐I was produced by another subset of pDCs where virus replication does not occur.^76^, ^77^ Besides the homotypic mechanism, the broader heterotypic interactions may play a more important role in antiviral immunity. It was observed that hepatitis C virus (HCV)‐infected cells trigger a robust IFN response in pDCs via a mechanism that requires active viral replication, direct cell–cell contact and TLR7 signalling.^78^ Moreover, the cooperative sensing between pDCs and multiple other infected cells such as cDCs and macrophages has been described.^53^, ^77^, ^79^ Finally, dependent on the pathogen, the close‐range interactions between pDCs and infected cells are mediated through multiple routes including exosomes,^78^ enveloped virions,^79^ LFA‐1‐mediated adhesion^60^ and the integrin‐mediated ‘interferogenic synapse’^80^ (reviewed by Reizis^54^).

Plasmacytoid dendritic cells also produce another class of potent innate antiviral interferons, the IFN‐λs or type III IFNs (IFN‐III) in response to viruses or synthetic TLR ligands.^81^ The IFN‐λs mainly serve as a first line of defence at the mucosal barrier, given that the IFN‐λ receptor (IFN‐λR), the specific receptor for IFN‐λ, is restrictively expressed on cells of epithelial lineage and on certain human leucocytes including pDCs and B cells.^82^ Importantly, IFN‐λs are observed to provide non‐redundant antiviral protection at mucosal sites including the respiratory and gastrointestinal tract.^83^, ^84^ Recently, IFN‐λs have also found to be involved in autoimmunity and antitumor immunity.^85^, ^86^ Besides, IFN‐λs could positively regulate pDC functions, including interferon‐dependent gene transcription,^87^ production of cytokines (including IFN‐I),^88^ maturation^81^ and survival.^88^ Therefore, during virus infection, the local defence by IFN‐λs at mucosal sites may enhance the subsequent systematic IFN‐I responses.

## pDCs and T‐/B‐cell responses

Antigen presentation by pDCs could context‐dependently lead to CD4^+^ T‐cell activation or tolerance induction. Upon *in vitro* activation by the influenza virus, human pDCs drive a potent Th1 polarisation.^2^ Meanwhile, CD40L‐activated human pDCs induce a strong Th2 response.^89^ Nevertheless, upon TLR7 activation or TGF‐β exposure, both human and mouse pDCs selectively promote a Th17 response.^90^, ^91^ When pDCs are either unstimulated or alternatively activated, they express the context‐dependent expression of indoleamine 2,3‐dioxygenase (IDO), inducible costimulator ligand, OX40 ligand (CD252), PD‐L1 and Granzyme B and induce regulatory T‐cell responses during viral infection, tumor and autoimmune disorders (reviewed by Swiecki and Colonna 2015).^46^

To identify the antigen‐presenting role of specific surface molecules expressed on pDCs, monoclonal antibodies (mAb) were used. By using a mouse model expressing human CD303 specifically in pDCs together with an anti‐CD303 mAb, it was confirmed that antigen delivery to pDCs through CD303 decreased effector CD4^+^ T cells and preserved Foxp3^+^ Tregs.^92^ Using similar methods, it was found that Siglec‐H‐mediated antigen delivery induced a hyporesponsive state of T cells *via* reducing expansion of CD4^+^ T cells and inhibiting Th1/Th17‐cell polarisation but not conversion to Foxp3^+^ Tregs.^93^ Moreover, antigen delivered to murine pDCs *via *BST2 in combination with TLR agonists as adjuvants is specifically presented by pDCs *in vivo* and elicits strong cellular and humoral immune responses.^94^

Besides antigen presentation to CD4^+^ T cells, it was also observed that both human and murine pDCs could cross‐present exogenous antigens to prime CD8^+^ T cells.^95^ Notably, murine pDCs acquire cross‐presentation capacity only when activated by TLR ligands, and mitochondrial reactive oxygen species is involved in the regulation of this process.^96^, ^97^ The recycling endosomes within pDCs facilitate CD8^+^ T cross‐priming by offering sites for loading peptide onto MHC class I, and subsequent cross‐presentation to CD8^+^ T cells.^95^ Moreover, it was recently observed that upon viral infection, pDCs would migrate to the CD8^+^ T‐cell priming sites in the lymph nodes in a strictly CCR5‐dependent manner, indicating a crosstalk between pDCs and CD8^+^ T cells which is yet to be investigated.^98^

A pioneering study showed that in response to influenza virus, human pDCs secrete IFN‐α and IL‐6, which mediate the differentiation of B cells into plasmablasts and the subsequent development into immunoglobulin (Ig)‐secreting plasma cells, respectively.^99^ Later, it was also observed that CpG‐stimulated human pDCs could induce plasma cell differentiation in naive and memory B cells in the absence of T‐cell help.^100^ Additionally, cell‐to‐cell contact also contributes to B‐cell proliferation and differentiation promoted by CpG‐activated human pDCs.^101^ Indeed, during virus infections such as human cytomegalovirus (HCMV)^102^ and rotavirus^103^, the activated pDCs play an important role in triggering B‐cell responses and enhance humoral immunity. Meanwhile, in many autoimmune diseases, the pDCs are abnormally activated and drive B‐cell responses involved in disease pathophysiology (introduced in the next section).^104^

It is noteworthy that some of the previously regarded capacities for pDCs to induce T‐/B‐cell responses (e.g. IL‐12 secretion and antigen presentation in part) may be attributed to the Axl^+^ DCs. ^16^, ^17^, ^18^ However, since the canonical IFN‐I‐producing pDCs retain antigen‐presenting capacity upon activation, their relationships with T/B cells require re‐evaluation.^22^, ^25^ Collectively, the correlations between pDCs and T/B cells play either beneficial or deleterious roles during infections and immune‐mediated diseases and warrant further investigation.

## pDCs in autoimmunity

The roles of pDCs in immune‐mediated diseases are summarised in Table 1. pDCs play an important pathogenetic role in SLE. Raised serum levels of IFN‐α and constitutive upregulation of IFN‐α‐inducible genes have been observed in SLE patients and are correlated with both disease activity and severity.^7^, ^105^ Importantly, during SLE and other autoimmune diseases, human pDCs sense the immune complexes formed by autoantibodies and nucleic acids mediated by FcγIIa (CD32A) or FcεRI expressed at the plasma membrane.^106^, ^107^ The immune complexes are then internalised through phagocytosis and delivered into phagosomal compartments, where TLR7 and/or TLR9 signalling initiates and finally leads to IFN‐α production.^71^, ^108^ In addition, pDCs are decreased in peripheral blood, activated and accumulated in the tissue lesions of SLE patients.^109^ Moreover, in SLE patients, pDCs promote plasmablast differentiation but fail to induce regulatory B cells.^104^ Consistent with the predominance of females among SLE patients, pDCs from females produce more IFN‐α upon TLR7 stimulation than those from males, probably due to both the effects of female sex hormone estrogens and the intrinsic X chromosome complement.^110^

**Table 1:** Role of pDCs in immune‐mediated diseases

Investigated disease	Role of pDC	Human/Mouse model & pDC depletion/modulation method	Possible mechanism	References
pDC in autoimmunity				
Systemic lupus erythematosus (SLE)	Disease initiation/promotion	**SLE patients** **Mouse models** BXSB lupus‐prone mice (BDCA2‐DTR: pDC depletion) B6.Nba2lupus model (BDCA2‐DTR) Tlr7 transgenic mice (Tcf4 haplodeficiency: pDC impairment) B6.Sle1.Sle3 multigenic SLE model (Tcf4 haplodeficiency)	Serum IFN‐α↑ IFN‐α‐inducible genes↑ Target organ migration pDC resistance to glucocorticoids pDC–neutrophil positive feedback Plasmablasts↑ aberrant regulatory feedback between pDC and Bregs	7, 105, 115, 116, 117
Systemic sclerosis (SSc)	Disease initiation/promotion	**SSc patients** **Mouse models** Bleomycin‐induced fibrosis model (CLEC4C‐DTR OR anti‐PDCA‐1 mAb: pDC depletion)	Target organ migration Fibrosis establishment and development IFN‐α and CXCL4 secretion	8, 121
Type I diabetes	Disease initiation/promotion	**Type I diabetes patients** **Mouse models** Non‐obese diabetic (NOD) mice (Tcf4 conditional knockout in CD11c^+^ cells: pDC impairment)	pDC recruitment to pancreatic islets IFN‐α secretion and insulitis induction	122, 123
Psoriasis	Disease initiation/promotion	**Psoriasis patients** **Mouse models** Xenograft model of human psoriasis (anti‐BDCA2 mAb: pDC impairment) DKO* mice (BDCA2‐DTR)	Skin migration IFN‐α production and activation/expansion of pathogenic T cells IL‐23 production	45, 124, 125
Rheumatoid arthritis (RA)	Disease prevention	**RA patients** **Mouse models** Serum‐transfer model of arthritis (IkL/L: pDC depletion)	Topical use of TLR7 agonist imiquimod cause Inflammation↓ bone destruction↓ IFN‐I signature induction	126
Inflammatory bowel disease (IBD)	**Controversy** Disease promotion	**IBD patients** **Mouse models** DSS‐induced acute colitis model (Siglec‐H DTR: pDC depletion)	pDC accumulation in the inflamed colonic mucosa Mobilisation of colitogenic phagocytes into the inflamed colon	127
IBD	**Controversy** Dispensable	**Mouse models** WASP‐deficient mice (Tcf4 haplodeficiency) IL‐10‐deficient mice (Tcf4^Fl/Fl^ mice: pDC depletion)	N/A	128
Atherosclerosis	**Controversy** Disease promotion	**Atherosclerosis patients** **Mouse models** Apolipoprotein E‐deficient mice (anti‐mPDCA1 mAb: pDC impairment) Ldlr^‐/‐^ mice (CD11c‐Cre × Tcf4^‐/flox^: pDC depletion)	Circulation pDC ↓ pDC detectable in human atherosclerotic plaques Proatherogenic T‐cell activation and lesional T‐cell infiltration	129, 130
Atherosclerosis	**Controversy** Disease prevention	**Mouse models** Ldlr^‐/‐^ mice (BDCA2‐DTR)	Aortic localised Treg generation via CCR9 and IDO‐1 expression on pDCs	131
pDC in alloreactivity				
Graft‐versus‐host disease (GVHD)	Sufficient but not necessary in inducing GVHD	**Mouse models** BALB/c → H2‐Ab1^‐/‐^B6 (graft: CD4^+^ T cell from BALB/c^+^ pDCs from WT B6 mice) C3H.SW→(CD11c‐DTR → B6) (anti‐BM stromal‐derived Ag Ab BST2: pDC depletion)	pDC maturation mediated by environment created by conditioning Prime of alloreactive T cells	133, 134
GVHD	Disease prevention	**GVHD patients: intestinal mucosa & skin** **Mouse models** BALB/c → C57BL/6 (graft: CD4^+^ T cell from BALB/c^+^ CCR9^+^ pDCs from Flt3L‐treated B6) C57BL/6 → B10.BR C57BL/6 → B6D2F1 (120G8 mAb: pDC depletion) C57BL/6 → NOD & C57BL/6 → BALB/c (donor Flt3L KO: exclude effects of Flt3L)	Engraftment enhancement Target organ recruitment Suppression of effector T‐cell responses Induction of Foxp3^+^ regulatory T cell IFN‐γ by donor T cells induces IDO secretion from donor pDCs → Treg/Th17↑	9, 10, 136, 139, 141, 143

DTR, diphtheria toxin receptor; Flt3L, Flt3 ligand; IDO, indoleamine 2,3‐dioxygenase; KO, knockout; mAb, monoclonal antibody.

A positive feedback loop between pDCs and neutrophils is abnormally upregulated during the SLE disease process. The circulating neutrophils in SLE patients may be primed *in vivo* by type I IFN excessively produced by pDCs and release more neutrophil extracellular traps (NETs) rich in antimicrobial peptides, self‐DNA, HMGB1 and oxidised mitochondrial DNA and will trigger pDC activation and excessive type I IFN secretion via the TLR9 pathway.^111^, ^112^, ^113^, ^114^

Genetic models have helped to understand better the pathogenic role of pDCs in SLE. Diphtheria toxin receptor (DTR)‐based transient depletion of pDC in lupus‐prone mice before disease onset resulted in amelioration of disease. Surprisingly, these effects were maintained even though pDCs later recovered, revealing the crucial role of pDC in disease initiation.^115^, ^116^ In addition, constitutive impairment of pDCs by monoallelic deletion of Tcf4 strongly reduced autoantibody production and all disease manifestations in two different spontaneous models of SLE.^117^ However, there remain caveats in genetic ablation or antibody‐mediated pDC depletion in these mouse models due to lower specificity and potency. For instance, besides pDCs, TCF4 is also an important regulator for germinal centre B‐cell and plasma cell development.^118^ Meanwhile, BST2 is also expressed on plasmacytes in steady state and on most cell types upon stimulation with IFN‐Is and IFN‐γ.^119^ In addition, the antibody‐mediated depletion could only exert transient effects and that certain genetic ablation methods such as the monoallelic deletion of Tcf4 could only induce partial reduction of pDCs.^117^ Techniques for *in vivo* depletion and functional modulation of pDCs, as well as their advantages and caveats, are well summarised by Reizis.^54^

Apart from SLE, pDCs were found to be implicated in several other autoimmune diseases. pDCs are responsible for most of the IFN‐α secretion in SSc patients and play a critical role during the process of fibrosis.^120^ Abnormally activated pDCs are infiltrated in the target organs such as skin and lung and found in bronchoalveolar lavage, and secrete IFN‐α and CXCL4 (both hallmarks of SSc), in both patients and mouse models.^8^ Moreover, in a SSc mouse model with bleomycin‐induced fibrosis, depletion of pDCs not only prevented disease initiation, but also ameliorated the established fibrosis.^8^, ^121^ In type I diabetes, pDCs are proportionally expanded in patients at disease onset.^122^ Indeed, pDCs are recruited and activated in the pancreas of non‐obese diabetic (NOD) mice, and TCF4 knockout in NOD mice has ameliorated insulitis and reduced diabetes incidence.^123^

In psoriasis, pDCs were recruited to the skin of patients via the chemerin/ChemR23 axis, became activated and produced IFN‐α early during disease formation.^45^, ^124^ Moreover, functional inhibition or early depletion of pDCs in a xenograft and a genetic model of psoriasis caused disease amelioration, respectively.^124^, ^125^

Plasmacytoid dendritic cells are not always disease‐promoting. In certain diseases, the role of pDCs may be protective. Enhanced pDC recruitment and activation to arthritic joints by topical application of the TLR7 agonist imiquimod ameliorated arthritis in a genetic mouse model.^126^ In addition, pDCs were found to infiltrate the intestinal mucosa of inflammatory bowel disease (IBD) patients; however, controversy remains over their exact role. Moreover, Arimura *et al.* ^127^ reported that pDC depletion using Siglec‐H‐DTR mice attenuated disease development in a chemically induced acute colitis model, while Sawai *et al.* ^128^ showed that monoallelic deletion of Tcf4 in two genetic models of IBD had no effect on disease development. pDCs have been shown to decrease in circulation and are detected in plaques during atherosclerosis in patients. However, conflicting results exist in *in vivo* experiments, as constitutive or transient depletion of pDCs prevented^129^, ^130^ or aggravated^131^ atherosclerosis in genetic mouse models. Therefore, the role of pDCs in certain autoimmune diseases may be spatially and temporally dependent.

## pDCs in alloreactivity

Alloreactivity is identified when immunocompetent T cells in the donated tissue (the graft) recognise the recipient (the host) as foreign and migrate to and attack the target organs in the immune‐compromised host.^132^ In clinical conditions, alloreactivity happens during GVHD, a major immunologic complication for patients who undergo allogeneic haematopoietic cell transplantation (allo‐HCT). A pioneering study showed that MHC‐expressing host pDCs alone were sufficient to prime alloreactive T cells and cause GVHD in a GVHD‐resistant mouse model, and pDC maturation was mediated by the inflammatory environment created by irradiation.^133^ However, *in vivo* depletion of host pDC, alone or together with cDC depletion, did not ameliorate murine GVHD.^134^ This is consistent with studies revealing that in allo‐HCT, many other cells, including donor antigen‐presenting cells (APCs) and recipient non‐haematopoietic APCs, are, with enough potency, sufficient to induce GVHD.^135^

The effects of pDC on the major target organ of aGVHD, the gastrointestinal tract (GI), have been under intensive investigation. Hadeiba and colleagues showed that CCR9^+^ pDCs were recruited to the intestines and attenuated aGVHD in a mouse model induced by allogeneic CD4^+^ T cells, probably *via* induction of Tregs.^136^ In addition, the pro‐inflammatory Th17 cells, together with pDCs, were upregulated in the intestinal mucosa of patients with aGVHD, as compared with patients without aGVHD.^9^ Moreover, this co‐upregulation of pDC and Th17 was also shown in the skin of aGVHD patients, as compared with healthy individuals.^10^

The content of pDCs within a graft, or the graft type, may affect GVHD severity. Unrelated BM allograft with a higher content of pDCs led to improved survival in GVHD patients as compared with grafts with lower pDCs.^137^ Relatively, in a MHC‐mismatched murine transplant model, recipients of Flt3L‐treated BM (containing a higher proportion of inactivated pDCs) had increased survival and decreased GVHD scores with fewer Th1 and Th17 polarised T cells post‐transplant as compared with recipients of unmanipulated BM.^138^ Interestingly, in a murine model of MHC‐mismatched transplantation, the 120G8 mAb‐mediated pDC depletion from BM grafts resulted in an acceleration of GVHD mortality while the pDC depletion from G‐CSF‐mobilised splenic grafts had no effect.^139^ This observation indicated the intrinsic difference between pDCs in BM and G‐CSF‐mobilised graft. Indeed, a subset of haematopoietic stem cells, the CD8^+^TCR^−^ ‘facilitating cells (FCs)’, has long been identified in murine BM but not in G‐CSF‐mobilised graft. FCs could enhance engraftment and promote transplantation tolerance *in vivo*.^140^ Further studies revealed that FCs contain a specific subset of pDC precursors which could attenuate GVHD in mouse models. These cells express Lin^−^CD11c^+^B220^+^PDCA1^+^ and predominantly develop into mature pDCs upon Flt3L activation.^141^, ^142^, ^143^ The GVHD prevention by these pDC precursors is probably mediated by IFN‐γ produced by donor T cells, which induce IDO synthesis by donor precursor pDCs and subsequent Treg generation in recipient mice.^143^ However, it is noteworthy that *in vivo* expansion by Flt3L is not pDC‐specific, as it would also induce development and proliferation of other cells (e.g. the CD3^+^ subset) within the FC population and exert anti‐GVHD effects.^144^

Post‐transplantation reconstitution of pDCs is predictive for subsequent GVHD risk. Patients developing aGVHD after myeloablative allo‐HCT were shown to have significantly lower numbers of both circulating cDCs and pDCs than non‐GVHD patients, and low DC counts were associated with severe aGVHD.^145^ Similar to myeloablative allo‐HCT, low pDC counts in patients receiving reduced‐intensity conditioning allo‐HCT were also correlated with severe grade II–IV aGVHD.^146^ Moreover, steroid treatment rapidly decreased pDC counts at all time points after transplantation.^147^ Nevertheless, recent studies in mouse models show that not only the quantity, but also the quality of DCs is altered during GVHD. On the one hand, GVHD impairs the murine pDC ability to prime the virus‐specific T cells.^148^ On the other hand, antigen presentation through MHC II is also impaired during aGVHD, leading to Treg deficiency and consequent chronic GVHD (cGVHD) in a pre‐clinical mouse model.^149^

## Targeting pDC functions in autoimmunity and alloreactivity

Given the pathogenetic role of pDCs in autoimmunity and alloreactivity, several molecules targeting pDCs have been assessed in clinical trials (summarised in Table 2). Recently, anifrolumab, the anti‐IFNAR1 mAb, has shown efficacy in moderate‐to‐severe SLE in a Phase III clinical trial,^11^ in which a BILAG‐based composite lupus assessment (BICLA) response occurred in 86 of 180 (47.8%) patients who received anifrolumab at week 52, compared with 57 of 182 (31.5%) of those who received placebo. This is the first Phase III trial confirming the efficacy of pDC‐targeting drugs in SLE. Sifalimumab^12^ and rontalizumab,^150^ the two humanised anti‐IFN‐α mAbs, have also shown efficacy in two Phase II clinical trials in moderate‐to‐severe SLE. Moreover, BIIB059, a humanised anti‐BDCA2 mAb, was shown in a Phase I trial to ameliorate skin lesions in SLE,^13^ and a Phase II trial is ongoing. Notably, since it was observed that pDCs depend more on the anti‐apoptotic protein BCL‐2 for survival as compared with cDCs, the BCL‐2 antagonists (e.g. the commercially available drug venetoclax) have been proven to selectively deplete pDCs, but not cDCs, *in vitro* and *in vivo*.^151^, ^152^ A Phase I clinical trial has confirmed the safety of venetoclax for SLE in female patients.^153^

**Table 2:** Clinical trials targeting pDCs in immune‐mediated diseases

Drug	Antigen	Format	Status	Disease	Results	References
Anifrolumab	IFNAR1	Blocking antibody	Phase III	SLE	Phase III: response at week 52: anifrolumab (47.8%) vs placebo (37.5%)	Phase III: 11; NCT02446912; NCT02794285
Anifrolumab	IFNAR1	Blocking antibody	Phase II	Lupus nephritis	Phase II ongoing	NCT02547922
Anifrolumab	IFNAR1	Blocking antibody	Phase II	Rheumatoid arthritis	Phase II ongoing	NCT03435601
Sifalimumab	IFN‐α	Blocking antibody	Phase II	SLE	Phase II met primary endpoint	12; NCT01031836; NCT00979654
Rontalizumab	IFN‐α	Blocking antibody	Phase II	SLE	Primary endpoint not met in Phase II, but disease improved in patients with low ISM scores	150
IFN‐α kinoid	IFN‐α	Vaccine	Phase II	SLE	IFN‐α kinoid was well tolerated in Phase I; Phase II ongoing	154
IFN‐α kinoid	IFN‐α	Vaccine	Phase II	DM	Ongoing	NCT02980198
BIIB059	BDCA2	Functional antagonist	Phase II	SLE	BIIB059 ameliorated skin lesion in Phase I; Phase II ongoing	Phase I: 13 Phase II: NCT02847589
DV1179	TLR7/9	Oligonucleotide inhibitor	Phase IIa	SLE	Primary pharmacodynamic endpoints not met in Phase IIa	–
PF‐06650833	IRAK4	Small‐molecule inhibitor	Phase II	Rheumatoid arthritis	Phase II completed and results submitted	NCT02996500
Venetoclax	BCL‐2	Small‐molecule inhibitor	Phase I	SLE	Venetoclax was well tolerated in Phase I;	153
CPG 52364	TLR7/8/9	Oligonucleotide inhibitor	Phase I	SLE	Phase I completed, no results posted	NCT00547014
VIB7734/MEDI7734	ILT7	Functional antagonist	Phase I	SLE, CLE, SSc, DM, PM, Sjogren's	Phase I completed, no results posted	NCT02780674 NCT03817424

BCL‐2, B‐cell lymphoma 2; CLE, cutaneous lupus erythematosus; DM, dermatomyositis; IFNAR1, type I IFN receptor subunit‐1; ISM, interferon signature metric; PM, polymyositis; SLE, systemic lupus erythematosus; SSc, systemic sclerosis.

Several new molecules are also progressing in the pipeline with the focus on depleting or inhibiting pDC. These molecules bind to surface receptors (such as BDCA2 or ILT7) or block endosomal TLRs, or TLR’s downstream signalling. Moreover, these molecules may not only inhibit the IFN‐I pathway, but also affect other pDC functions such as the production of TNF‐α, IL‐6 and chemokines and antigen presentation.^154^ Some of them have been assessed in pioneering clinical trials (Table 2).

Arsenic trioxide (As_2_O_3_), a well‐established drug for acute promyelocytic leukaemia, was observed to have therapeutic potential in pre‐clinical mouse models of SLE,^155^ SSc^156^ and sclerodermatous GVHD,^157^ with unknown mechanism.^158^ Our group has recently offered a potential explanation by demonstrating that clinically relevant concentrations of As_2_O_3_ preferentially block IFN‐α secretion from pDCs through IRF7 inhibition and also impair the capacity of pDCs to induce T‐/B‐cell responses.^159^ We are currently running a prospective multicentre clinical trial testing As_2_O_3_ in the setting of cGVHD (ClinicalTrials.gov identifier: NCT02966301).

## Concluding remarks

The heterogeneity of pDCs has been revealed recently, especially in the last five years. With the development of single‐cell analysis, the previously identified pDC population has been separated into Axl^+^ DCs and canonical IFN‐I‐producing DCs. Therefore, although Axl^+^ DCs constitute only a small proportion (10–15%) of the traditionally defined pDCs,^20^, ^21^, ^22^ this putative DC subset must be independently investigated in future studies of pDCs. Moreover, recent evidence has indicated that both the Axl^+^ DCs and canonical DCs are indeed heterogeneous at both phenotypic and genetic levels, prompting us to study pDCs with more precise and comprehensive techniques in the future.^21^, ^22^, ^25^

Plasmacytoid dendritic cells could be derived from both lymphoid and myeloid origins. However, recent studies have provided strong evidence that the lineage imprinting of pDCs happens early before the emergence of the myeloid/lymphoid progenitors, and probably at the level of haematopoietic stem cells.^26^, ^29^ These observations have challenged the current theory of leucocyte development and indicated that the previously regarded ‘homogenous’ progenitors are indeed heterogeneous. Moreover, the capacity of freshly isolated pDCs to differentiate into cDC‐like cells discovered in both humans and mice reveals an intrinsic plasticity of differentiated pDCs.^35^, ^160^, ^161^ To answer these questions requires a better characterisation of pDC fate and poses important challenges for future studies.

The exact roles of pDC in most autoimmune diseases are still far from elucidation. Positive results for the anti‐IFNAR1 mAb anifrolumab in a Phase III SLE trial have provided encouraging evidence for the use of pDC‐targeting drugs in SLE. In addition, in alloreactivity, pDCs may play either a protective^136^, ^143^ or deleterious role,^9^, ^10^ making the effects of pDC depletion unpredictable. Collectively, more studies must be done to understand more fully the biology of pDCs in the initiation/development of autoimmunity and alloreactivity, and novel pDC‐targeting modulation drugs are to be expected.

## Conflict of interest

The authors declare no conflict of interest.
